# ML-MEDIC: A Preliminary Study of an Interactive Visual Analysis Tool Facilitating Clinical Applications of Machine Learning for Precision Medicine

**DOI:** 10.3390/app10093309

**Published:** 2020-05-09

**Authors:** Laura Stevens, David Kao, Jennifer Hall, Carsten Görg, Kaitlyn Abdo, Erik Linstead

**Affiliations:** 1 Department of Cardiology, University of Colorado Medical School, Aurora, CO 80045, USA;; 2 Cardiovascular Medicine, Institute for Precision Cardiovascular Medicine at the American Heart Association, Dallas, TX 75231, USA;; 3 Electrical Engineering and Computer Science, Chapman University, Orange, CA 92866, USA;

**Keywords:** data science, interactive visual analysis, data-driven medicine, machine learning, cloud computing

## Abstract

Accessible interactive tools that integrate machine learning methods with clinical research and reduce the programming experience required are needed to move science forward. Here, we present Machine Learning for Medical Exploration and Data-Inspired Care (ML-MEDIC), a point-and-click, interactive tool with a visual interface for facilitating machine learning and statistical analyses in clinical research. We deployed ML-MEDIC in the American Heart Association (AHA) Precision Medicine Platform to provide secure internet access and facilitate collaboration. ML-MEDIC’s efficacy for facilitating the adoption of machine learning was evaluated through two case studies in collaboration with clinical domain experts. A domain expert review was also conducted to obtain an impression of the usability and potential limitations.

## 1. Introduction

Machine learning continues to show great promise in its ability to extract new biomedical insights or predict clinical outcomes, which requires medical researchers to be able to understand and interpret machine learning models in a clinically translatable manner. The translation of these models is often challenging due to the experience needed for clinical interpretation, validation of machine learning models, coding expertise among clinical researchers, security associated with biomedical datasets, and accessibility of computational tools to run machine learning pipelines in clinical settings [1–5].

The use of point-and-click, interactive tools that are accessible in secure internet access settings can be an effective medium to engage medical researchers in data analytics because they provide potential solutions to challenges associated with the clinical adoption of machine learning. Reactive and visual point-and-click tools limit the need for rewriting analyses when parameterizing and optimizing machine learning pipelines as well as enable users to visually compare and interpret multiple models without requiring coding expertise [6,7].

We present Machine Learning for Medical Exploration and Data-Inspired Care (ML-MEDIC), a point-and-click, interactive tool with a visual interface for facilitating machine learning and statistical analyses in clinical research. ML-MEDIC’s efficacy for facilitating the adoption of machine learning was evaluated through two case studies in collaboration with clinical domain experts. A domain expert review was also conducted as a preliminary study to obtain preliminary evidence regarding tool usability and potential limitations.

## 2. Materials and Methods

### 2.1. Data

To demonstrate the utility and usability of ML-MEDIC, we constructed two case study exercises. Longitudinal study data from the Framingham Heart Study (FHS) was used for case study 1. A pooled dataset containing data combined from the Framingham Heart Study (FHS), Cardiovascular Health Study, Multi-Ethnic Study of Atherosclerosis (MESA), and Atherosclerosis Risk in Communities Study (ARIC) was used for case study 2. The study design, response rates, and methodologies for each study are reported elsewhere [8–10].

### 2.2. Specified User Tasks

We iteratively consulted domain experts in the medical, statistical, and informatics fields to determine the most appropriate user tasks ML-MEDIC needed to address to aid in facilitating the adoption of machine learning in medicine. We consulted a cardiologist with extensive predictive analytics experience as well as a data science expert with experience in clinically applying contemporary statistical and machine learning methods. Additional cross-domain experts were consulted as needed, including an applied behavior analyst, a human–computer interaction (Graphical user interface/visualization) specialist, an epidemiologist, and a clinical psychologist.

In collaboration with the domain experts, we identified the following user-specified tasks that ML-MEDIC needed to facilitate the clinical application and adoption of machine learning:

1. Task 1: A tool that facilitates interpretable machine learning, through multiple model comparison, possible understanding of variables input into a model, and validation of machine learning methods with traditional statistical models. The clinical experts we spoke with all expressed concerns regarding the “black-box” nature of machine learning algorithms and the potential for spurious applications and conclusions. They expressed the need to be able to understand what input variables were most important to a model’s predictive output and the ability to compare and validate this with other commonly used statistical methods. When asked to consider trade-o s such as improved performance with “black-box” methods vs. worse performing methods with clearer interpretability, the domain experts suggested a preference for understandability initially and suggested that they would be more likely to use less interpretable methods only after evaluating more familiar, explicable methods.
2. Task 2: Ability to implement machine learning analyses without learning new coding languages, accessing new software, and collaborating on platforms without worrying about data security. The medical, data science, and statistical experts we interviewed expressed that software and pipelines designed for machine learning and statistical modeling require collaboration from multiple domains to be employed successfully and to meet data security standards. The collaborative nature of these analyses often means installing and running computational pipelines on multiple computers. Accessing software and pipelines in multiple environments often requires permission and assistance from one or multiple institutions and can often lead to challenges with accessibility, interoperability, and reusability. Even with correct versioning and identical data, differences in local hardware and operating systems can prevent interoperability and reproducibility.

### 2.3. Tool Design

The overall design goal for ML-MEDIC was to provide a point-and-click user-friendly tool that could facilitate the adoption of machine learning and data science in medical settings. We integrated visual and reactive computational approaches into the design to create an easy-to-use application that can engage domain experts, independent of their coding expertise. We chose R/Shiny as a framework to implement ML-MEDIC because of its open-source nature, interactive capabilities, and ability to re-compute calculations as necessary based on user input [11].

We deployed ML-MEDIC in a cloud environment for accessibility and reproducibility. Cloud-based environments have recently been developed to improve data access, sharing, and collaboration, as well as provide means for managing computational load and challenges associated with storing and analyzing big data [12,13]. We chose to deploy ML-MEDIC in an Amazon Web Services cloud environment built by the American Heart Association (AHA) for Precision Cardiovascular Medicine. The Precision Medicine Platform, a secure, Health Insurance Portability and Accountability Act (HIPPA)- and Federal Risk and Authorization Management Program (FedRAMP)-certified cloud-based ecosystem, is an interactive environment for facilitating data sharing, collaboration, and power computing. The platform is comprised of common machine learning tools and biomedical datasets, which enables researchers to easily store, analyze, and build analysis pipelines collaboratively without worrying about data security through the use of workspaces. Workspaces enable users to share data privately or with the public, perform analytics, and securely access and reuse analytic pipelines virtually [14].

Given input from clinical domain experts, we chose to focus on supervised machine learning methods for ML-MEDIC due to their ability to provide more clinically relevant results, and the domain experts identified noted supervised models as some of the models they most frequently rely on for their data analysis. However, the ML-MEDIC was designed in a modular manner to facilitate the addition of other models, including unsupervised ones. The machine learning algorithms were implemented using Caret, with parallelized options to optimize the computing time and improve reactivity [15–18].

**Figure 1.**
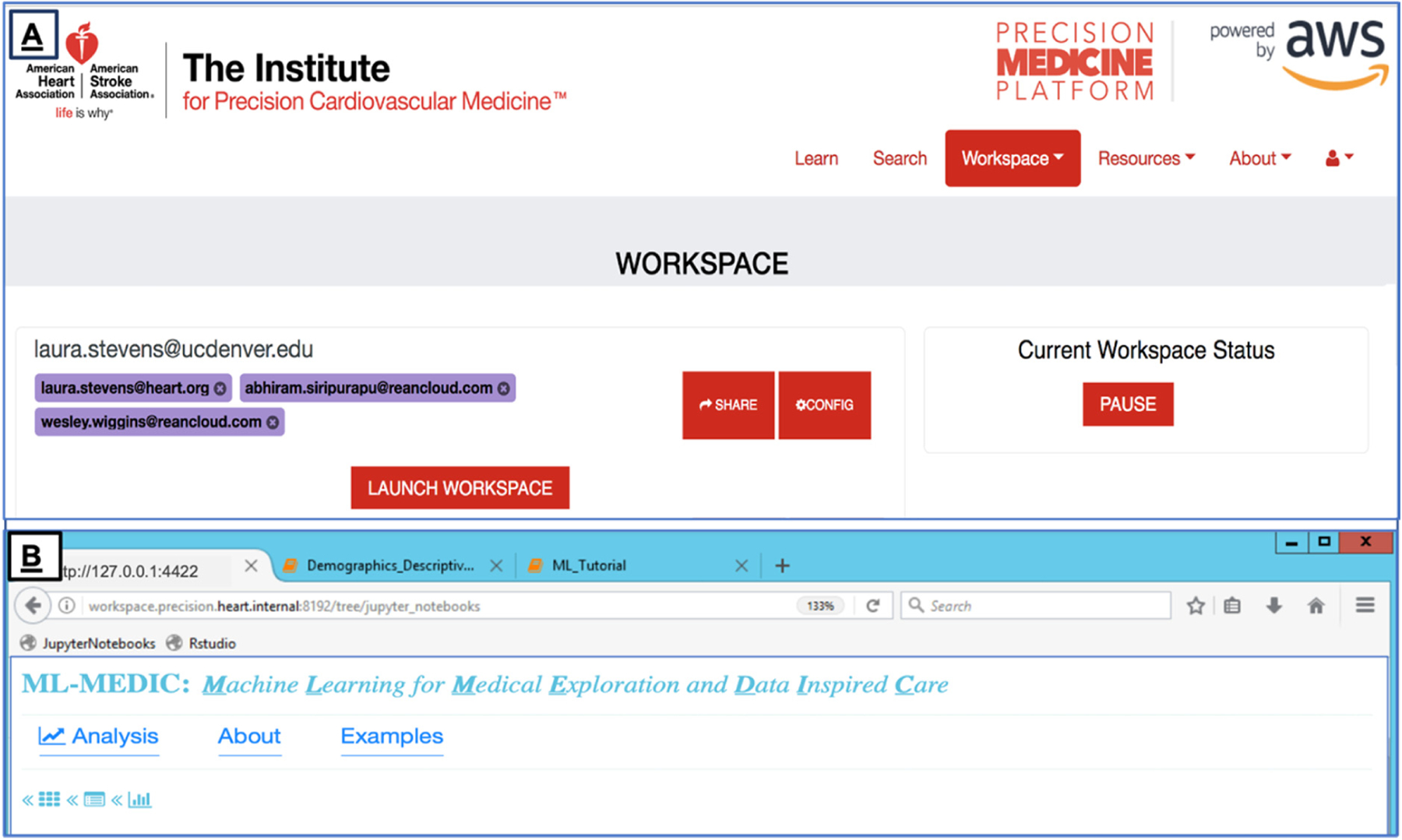
The Precision Medicine Platform. (**A**) The landing page of the workspace for the cloud-based, High Performance Computing-enabled platform ML-MEDIC in the Precision Medicine Platform that supports multiple collaborators (red box) to work in a secure virtual environment. (**B**) ML-MEDIC deployed in the platform, enabling reproducibility and accessibility. ML-MEDIC, Machine Learning for Medical Exploration and Data-Inspired Care.

We deployed ML-MEDIC in the Precision Medicine Platform to limit challenges associated with reusing or installing the tool on multiple computers, providing secure internet access to the tool, and allowing users to collaborate in real time (Figure 1). The AHA’s HIPPA- and FedRAMP-certified Precision Medicine Platform facilitates reusability and reproducibility by creating a virtual environment and a workspace that segregates the computation from the underlying hardware and host’s operating system [14,19]. The platform currently uses Chef to ensure the correct versioning and configurations are applied consistently at any scale [20]. The cloud-based ecosystem also includes a data marketplace for data contributors to securely share datasets and analytics with the public, even if data is codified in Data Use Agreements (DUAs). A variety of datasets are accessible through the platform, and users can share analytic pipelines, results, and code through traditional methods such as GitHub, as well as the through the platform itself [21–23].

**Figure 2.**
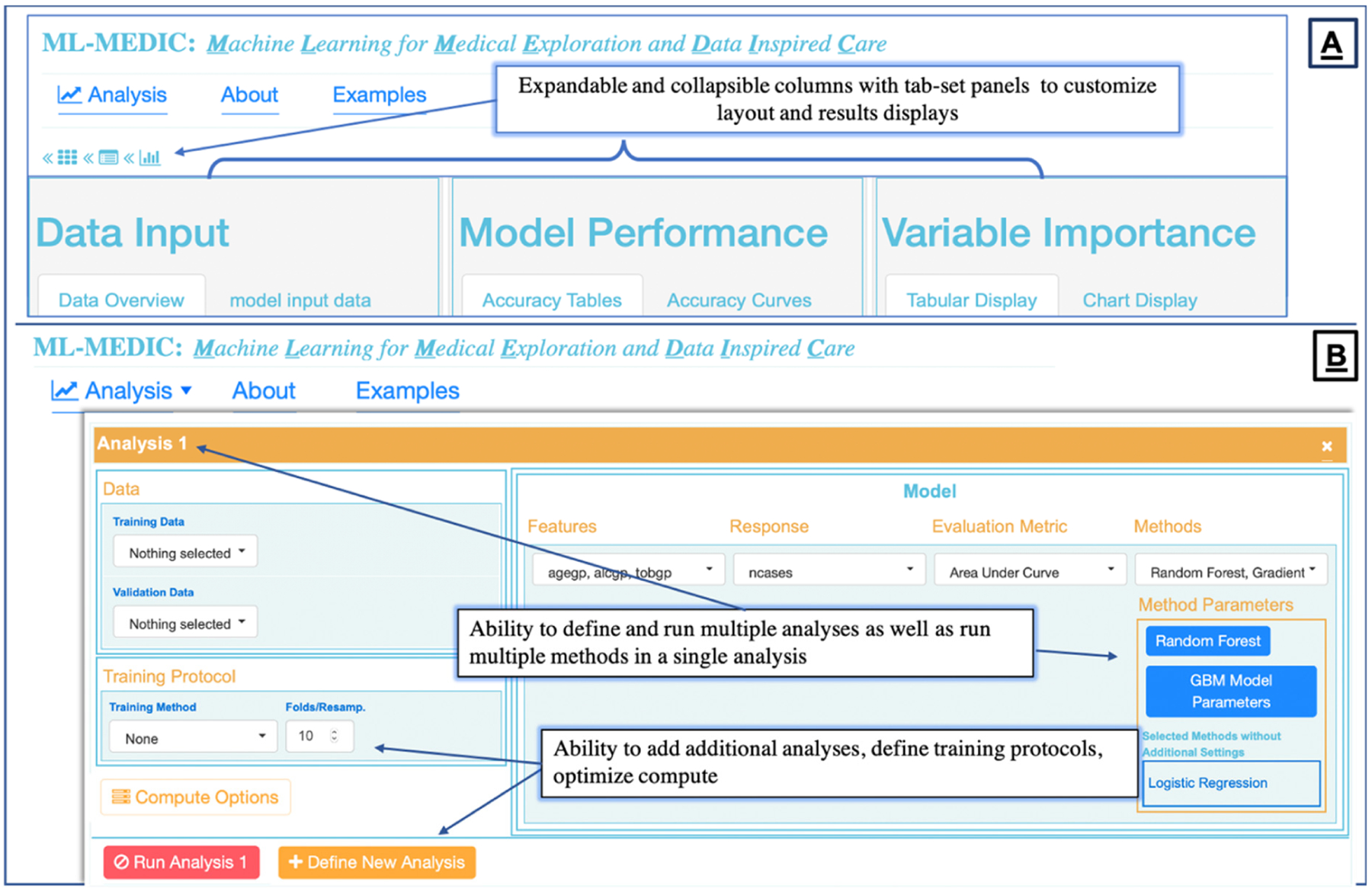
(**A**) Overall layout of ML-MEDIC. Expandable and Collapsible panels combined with tabular and graphical displays of results allows users to customize outputs and necessary visualizations at any point in the ML workflow. (**B**) Analysis dropdown menu allows for users to define one or multiple analyses and customize data input, training protocols, evaluation metrics, and run multiple ML and statistics models at once.

**Figure 3.**
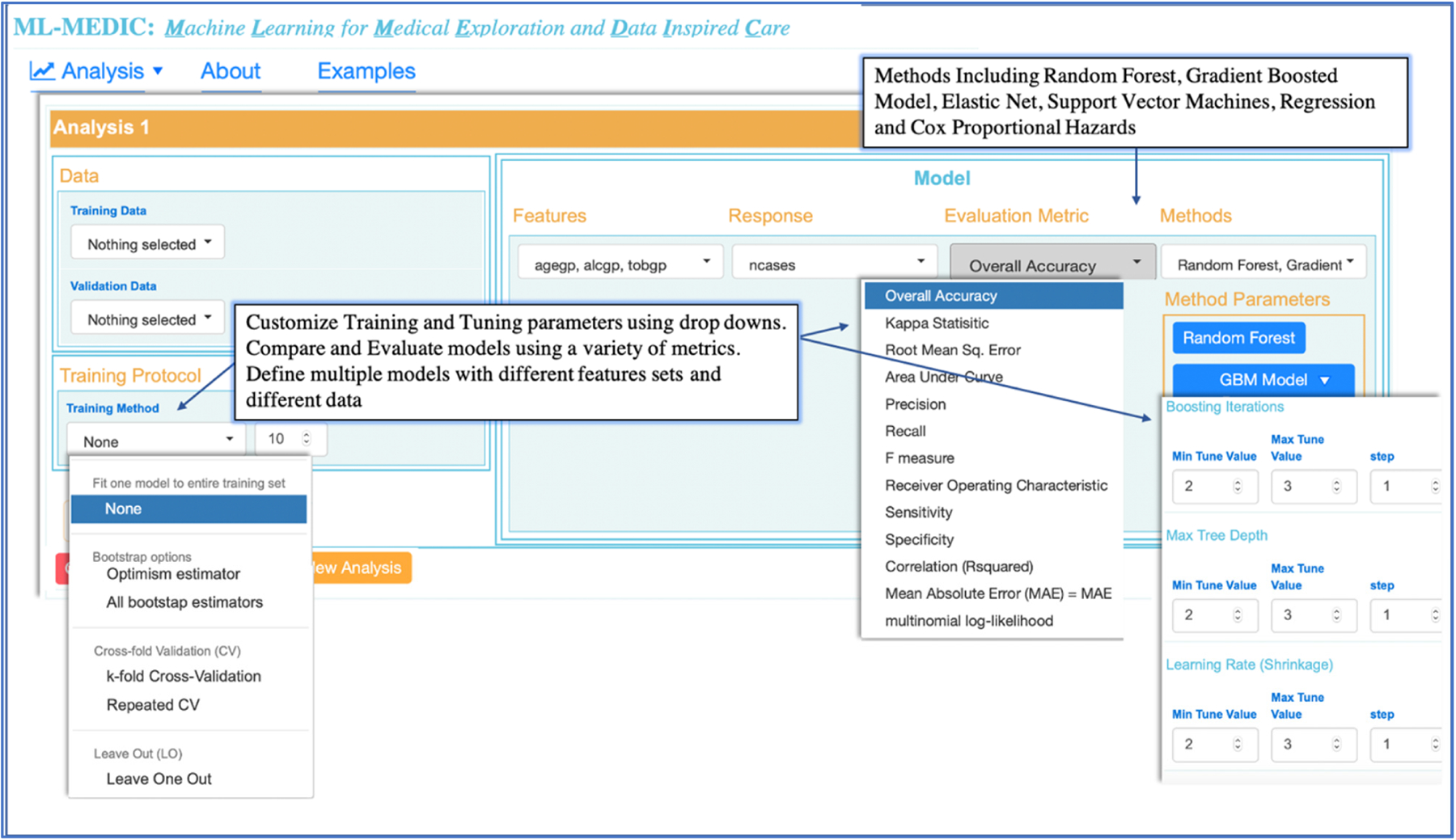
Overview of menus and options available for an analysis. Training protocols include bootstrap, cross-validation, and hold out methods. Accuracy metrics available for classification as well as numeric or multiclass models. Each available method contains a custom menu dropdown to define tuning parameters, with default values set based on standard practices and the data loaded for analysis. Each method or parameter available is defined in a modular manner to facilitate addition of other methods and features.

To create an “easy-to-use” interface, we structured the menu design to reflect the flow of a typical predictive analysis to align the interface design with the steps one would expect to implement when building a predictive model [5,24]. We identified these steps to be: (1) loading data; (2) building one or multiple models for comparison, including the ability to build the same model on two different datasets; (3) setting optional training and control parameters; (4) testing and evaluation (Figures 2 and 3).

### 2.4. Visual Analytics and Computational Approach—Task 1

**Figure 4.**
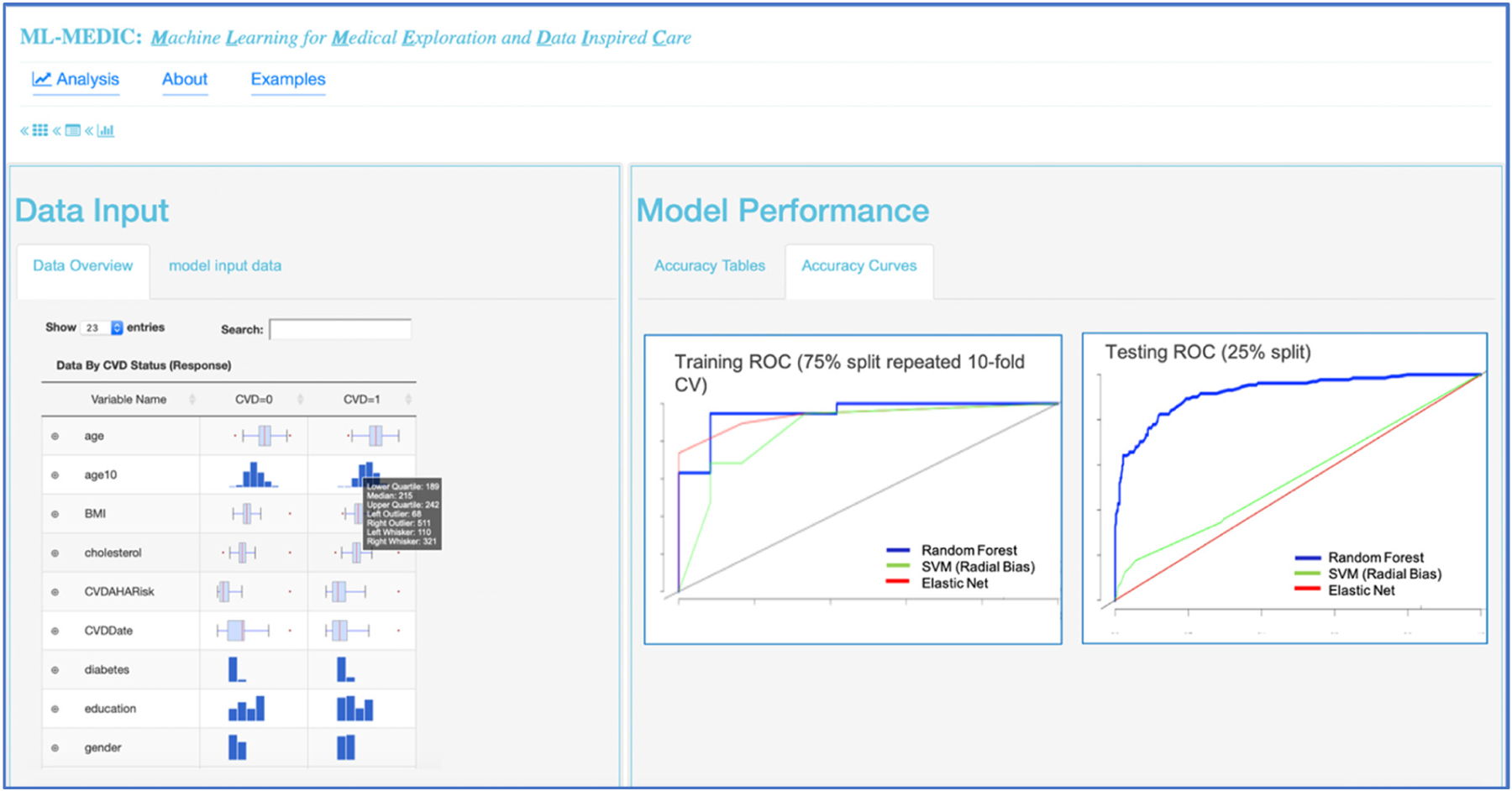
Results from case study 1 using ML-MEDIC. Three machine learning models were conducted to assess their potential to predict cardiovascular disease (CVD) using factors in the American Heart Association (AHA)/American College of Cardiology (ACC) CVD risk score. For comparison, random forest (RF), support vector machine (SVM), and elastic net plots are shown. Data tables showing distributions split by the outcome CVD (response variable) are on the left. ROC curve of training data (75% 10-fold repeated CV) compared to ROC curve of testing data on the left.

**Figure 5.**
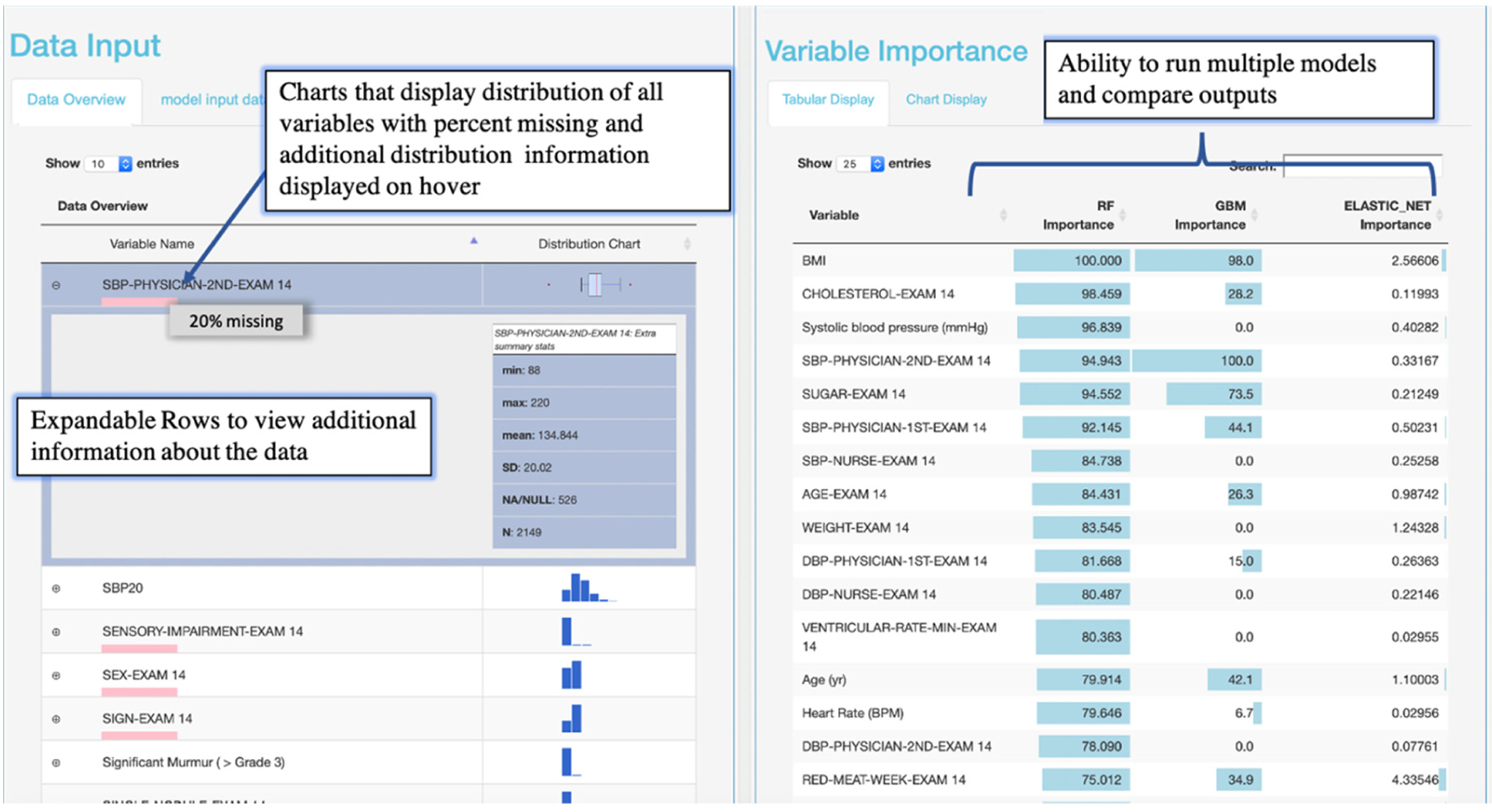
Results from case study 2. Tabular views of the data with expandable rows to obtain additional information relating to variables in the data and variables with a given importance from machine learning output (left). Variable importance tabular layout showing importance metrics scaling from 1 to 100 for all variables input into the model. Weekly red meat consumption is in the topmost important among other known variables.

Interactive visualizations were implemented to facilitate model implementation and interpretation. Interactive tabular and graphical summaries displaying the loaded data’s variable count and total sample size, and each variable’s distribution and missingness were implemented to visually support the user in setting the outcome variable and variables input into the model (steps 1 and 2) Additional tabular and graphical summaries are displayed to show the distribution of each variable when a response variable has been set (Figure 4). Default values for model parameters, cross-validation metrics, and splitting the data into training and testing sets were added to facilitate model implementation (steps 2 and 3). Tabular and graphical displays for model performance and variable importance were added to provide insight into how each model made predictions (step 4) (Figures 4 and 5). Graphical and tabular displays are displayed side by side, with custom settings for the user. The layout and display were added to facilitate the comparison of multiple models (Figures 3–5).

Literature reviews combined with the input from domain experts drove the selection of the machine learning and statistical methods available in ML-MEDIC [25,26]. Random forest (RF), elastic net, support vector machines (including polynomial, linear, and radial kernels), and gradient boosted modeling (GBM) (boosted classification and regression) (Figure 3) were selected as optional machine learning methods. We confined the methods available to models that were suggested by domain experts to be easily received by clinicians, could provide variable importance measures, and could serve as an intuitive gateway to ML from traditional regression and statistical models. To be able to compare machine learning methods to traditional statistical models, Cox proportional hazards, linear, and logistic regression were included in the models available for selection.

**Figure 6.**
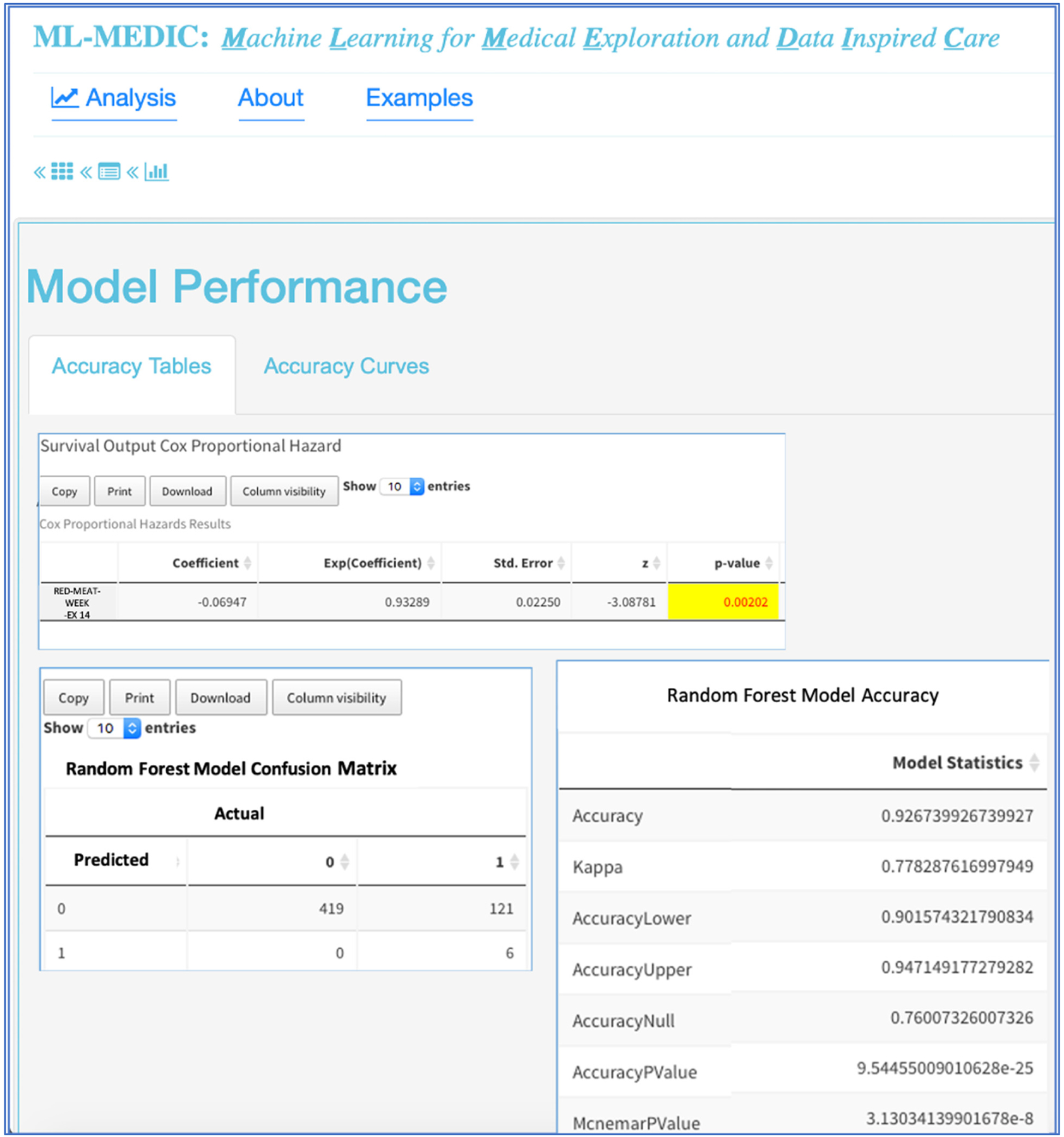
Results from case study 2. Collapsible views enable expansion and custom displays when comparing model outputs. The importance of red meat consumption in predicating heart failure is validated in Cox proportional hazards model, and random forest accuracy for the model with red meat and known heart failure risk factors are displayed.

A ROC curve with AUC and tables displaying performance metrics from both training and test datasets are displayed to assist users in comparing and evaluating models. Performance metrics include accuracy, AUC (c-statistic), and kappa for classification analyses, with options to include additional metrics such as precision, recall, F1, specificity, and multinomial log likelihood. For regression modelling, metrics such as mean absolute error, root mean square error, and others, are available for display (Figures 3 and 6). For all methods in which variable importance is available (RF, GBM, elastic net, CPH, regression), a user-specified view of feature importance is displayed, and the user can choose a tabular or interactive bar chart display (Figure 6). If Cox proportional hazards, linear, or logistic regression is selected as a model, a forest plot or tabular display of the results is included to compare with the variable importance of other models.

### 2.5. Visual Analytics and Computational Approach—Task 2

Dropdown menus, auto-completion, checkboxes, and buttons were used to create a reactive point-and-click interface consistent with the design and allow users to specify parameters and display preferences without coding expertise (Figures 2 and 3). Verification hints and error messages were implemented to reduce the error rate while enabling collaborators of all levels of coding expertise to iteratively adjust and implement machine learning. The ability to save a summary report of the data, model, and results was enabled to support publication or share pipelines with users without data access.

## 3. Results

### 3.1. Domain Expert Reviews

Domain expert reviews provide an impression of a visual interface’s usability and identify pitfalls without having to perform a full-fledged user study [27]. To initially evaluate and improve ML-MEDIC, we asked three clinical researchers to be involved in an initial review. After a short introduction, each clinician was paired with the tool developer to use the tool and explore machine learning applications in the context of their research. Five additional clinical researchers were given a demonstration of the tool using data and methods to evaluate its potential to facilitate machine learning adoption. All clinicians were asked to (1) give comments regarding their data, research context, and overall impression of the tool, and (2) rate the tool (“positively”, “neither-nor”, or “negatively”) with respect to ease of use, layout, and likeliness to use.

The overall opinion of the domain experts was positive regarding ease of use, layout, and likeliness to use, including comments such as “The general design is easy to follow, the tables and graphics are well done, and is useable without having to code” and “It runs very fast, and is something I could use when collaborating with colleagues who have limited machine learning experience or are just getting started.” Some experts explicitly appreciated the collaborative aspect and the compute power of ML-MEDIC implemented in the Precision Medicine Platform and liked not having to worry about installation or reusability. Many experts also appreciated the open-source nature of the code, with comments such as “this tool is easy enough to use and has the methods I need, to perform analyses without licensed tools like SAS or STATA” and “This is perfect for teaching machine learning”.

### 3.2. Case Study 1: Evaluate the Predictive Power of Various Machine Learning Methods to Predict Cardiovascular Risk

In 2013, the American College of Cardiology (ACC) and the American Heart Association (AHA) published a Cox proportional hazards model to predict patient cardiovascular (CV) risk in primary care. This model is one of the first cardiovascular risk models to include separate models based on both gender and race, comprised of a specific Cox proportional hazards equation for African American Men and African American women, Caucasian Men and Caucasian Women. Since the model’s release, the use of different statistical methods and cross-validation techniques have shown the potential to increase the accuracy of risk estimates.

Collaborating with a data scientist, biostatistician, and cardiologist, we used pair analytics methodology to conduct this study, in which a domain expert and a tool developer collaboratively analyzed the data [28]. The overall analysis session lasted three hours. Given research suggesting net elastic models with cross-validation showed improved accuracy for predicting cardiovascular disease risk, the cardiologist wanted to investigate the performance of a random forest model and GBM compared to elastic net, using 10-fold, repeated, cross-validation. Using the tabular views of the data, each expert could easily see summary statistics of cases and controls (Figure 5). Setting each model and the cross-validation parameters, the input and response variables did not require any coding expertise. The total compute time to run all three models was less than ten minutes, which allowed all collaborators to reactively compute and recompute various models. Using the ROC curve, the clinician could easily compare each model’s predictive performance to the performance of the elastic net, and the accuracy table could be used to numerically evaluate the performance. The random forest model resulted in the highest performance of the three models (Figure 3).

### 3.3. Case Study 2: Determine Dietary Factors Important in Predicting Congestive Heart Failure (CHF), and Whether They are Significant for Predicting CHF Risk

We collaborated with a cardiologist with experience in clinical research and data science to conduct case study 2. His research was focused on analyzing cardiovascular disease (CVD) datasets to identify patterns in clinical and genetic data to treat and prevent congestive heart failure (CHF) and stroke. The cardiologist was interested in using ML-MEDIC to perform feature selection using data from the Framingham Heart Study (FHS) and focused on phenotypes related to diet and well-known risk factors such as age, cholesterol, blood pressure, and body mass index [8]. Again, pair analytics methodology was used, and the analysis session lasted 2 hours. The security of the Precision Medicine Platform meant that the data could be uploaded and used with ML-MEDIC without having to install the code.

To begin the analysis, the cardiologist input all variables into a random forest model, elastic net model, and a gradient boosted model. The cardiologist used the model comparisons layout and variable importance to evaluate important phenotypes in predicting CHF (Figure 5). He used the importance plots and his domain expertise to interactively select features and identified known risk factors, but also identified unknown factors such as red meat and consumption, which was significantly associated with a decreased risk in all models. The reactive nature of the tool enabled the cardiologist to save the importance output, rerun the models with a select set of features from the importance plots, and add a Cox proportional hazards model to validate the machine learning variable importance of red meat in the survival of CHF. First, he started with univariate regression and then multivariate regression, adding known risk factors from the AHA/ACC CVD risk model. He found that red meat was significant in univariate and multivariate survival models for CHF and that it was associated with a decreased risk of CHF [29].

## 4. Discussion

The key findings from the development and analysis of ML-MEDIC are as follows:

- User interaction-based tools that do not require clinicians and biomedical experts to learn coding will facilitate machine learning due to ease of use.
- Initially learning a subset of methods minimizes the learning-curve, especially when more explainable methods are used, could facilitate the adoption of machine learning and could aid in clinician’s understanding of when certain machine learning methods can be applied as well as introduce trade-o s between interpretability and performance.
- Ability to access tools in secure internet access settings can eliminate challenges relating to reusability and access.
- A tool such as ML-MEDIC may provide a great educational resource for introducing data science and machine learning to clinical researchers.

In summary, all clinicians positively expressed the likelihood of using ML-MEDIC or similar pipelines and tools developed through resources such as Shiny in R or Django in Python to facilitate machine learning due to ease of use. They confirmed that a limited set of models, with references for other resources, were effective for minimizing the learning curve and facilitating decisions when initially learning how to implement machine learning methods. All clinicians appreciated the layout, tabular views, and charts when analyzing and comparing models and suggested that the reactive nature of the tool aided in collaboration and exploration. Many clinicians with private datasets appreciated the security and accessibility the Precision Medicine Platform provided and the use of high performance computing to improve compute time. Given certain requests for the ability to install ML-MEDIC on a personal computer or institutional servers, the code is available on GitHub (https://github.com/lmrstevens3/Clinical_Data_in_Shiny.git) and the learn page of the Precision Medicine Platform. Initial feedback on ML-MEDIC’s functionality and usability from the domain experts involved in the case studies was positive. An expert from case study 1 explained that “the ability to isolate meaningful features intuitively allows users to quickly prototype more advanced ML models using ML-MEDIC. As a result, clinicians can bring to bear the full power of predictive analytics on their datasets with only a lay understanding of data mining algorithms.” The cardiologist from case study 2 concluded that “ML-MEDIC easily demonstrated machine learning models could identify known risk factors associated with clinical outcomes, but also identify novel potential risk factors for better prediction of complex disease risk.” He specified that “easy-to-use and interactive tools would most likely be received well from both bioinformatics, statistical, and clinical audiences if they could confirm known findings while shortening the time for model building and evaluation”.

These preliminary results regarding the usability of ML-MEDIC and its ability to facilitate learning and implementing machine learning in clinical analyses suggest the generalizability of the tools to other clinical domains beyond cardiology. During this review, researchers in psychology and other clinical domains were consulted, and they affirmed that the tools could expand beyond cardiology disciplines. Furthermore, researchers suggested that this type of tool could be very beneficial in educational settings, such as for undergraduates first learning machine learning or clinicians and biomedical students learning data science. Currently, a variety of tools offer machine learning capabilities, yet the complexity and diverse functionality of these tools can come at the cost of overwhelming users with options and potentially hinder adoption of machine learning. Tools that favor ease of use over diverse functionality and application, such as ML-MEDIC, limit the barrier of entry and create a more gradual learning curve and can potentially act as a bridge to tools with fully customizable user-specified settings and complex functionality.

We limited ML-MEDIC’s machine learning methods to supervised learning due to the ability of supervised learning to generally provide more clinically relevant results and the input from the medical experts interviewed. The domain experts interviewed affirmed that comparing predictive supervised machine learning models to statistical models such as survival or logistic regression was most likely to facilitate the adoption of machine learning. Currently, ML-MEDIC does not support interactive data preparation for machine learning analyses. A potential next step would be to provide interactive methods for data preparation prior to performing machine learning. In addition to expanding the methods available, features further facilitating the interpretation and evaluation of models, such as adding a precision-recall curve to the current tabular display, or incorporating more model specific visualizations, such as graphical tree displays for decision-tree-based methods, can be explored [30]. The current evaluation of ML-MEDIC is limited to more of a qualitative assessment of the benefit of GUI-based, user-interactive tools by the clinical community to facilitate and improve the use of ML in clinical applications. Further testing and quantitative analysis is needed to assess resources such as ML-MEDIC and the potential for use in clinical decision support and various clinical research applications [31].

## 5. Conclusions

The development and implementation of effective machine learning applications in medicine often require collaborative effort across multiple disciplines. Part of facilitating the adoption of machine learning in medicine is lowering barriers associated with sharing and reusing data and analyses as well as supporting the collaborative efforts needed [19,32]. Here, we have presented ML-MEDIC, a user-interactive tool implemented in a secure, cloud-based environment, in order to enable accessibility and reusability, and support secure data sharing. This allows non-technical users to access machine learning tools and perform analyses, while being able to build upon already developed pipelines without worrying about installation or having to code. As the era of big data and cloud computing continues to evolve in medicine, interactive computing for predictive analytics and big data analysis will specifically be needed to connect expertise across multiple domains, encourage collaborations, and aid in reproducibility.
